# MiR-181b regulates cisplatin chemosensitivity and metastasis by targeting TGFβR1/Smad signaling pathway in NSCLC

**DOI:** 10.1038/srep17618

**Published:** 2015-12-01

**Authors:** Xiaoyuan Wang, Xuesong Chen, Qingwei Meng, Hu Jing, Hailing Lu, Yanmei Yang, Li Cai, Yanbin Zhao

**Affiliations:** 1 Department of Internal Medical Oncology, Harbin medical University Cancer Hospital, Harbin, Heilongjiang Province, China; 2 Cancer Research Institute, Harbin Medical University, Harbin, Heilongjiang Province, China

## Abstract

MicroRNAs (miRNAs) have been identified as important post-transcriptional regulators involved in various biological and pathological processes of cells, but their underlying mechanisms in chemosensitivity and metastasis have not been fully elucidated. The objective of this study was to identify miR-181b and its mechanism in the chemosensitivity and metastasis of NSCLC. We found that miR-181b expression levels were lower in A549/DDP cells compared with A549 cells. Functional assays showed that the overexpression of miR-181b inhibited proliferation, enhanced chemosensitivity to DDP, attenuated migration and metastatic ability in NSCLC cell lines *in vitro* and *in vivo*. TGFβR1 was subsequently identified as a novel functional target of miR-181b. TGFβR1 knockdown revealed similar effects as that of ectopic miR-181b expression, whereas overexpression of TGFβR1 rescued the function of miR-181b-mediated growth, chemosensitivity and metastasis in NSCLC cells. In addition, miR-181b could inactivate the TGFβR1/Smad signaling pathway. We also observed that decreased miR-181b expression and increased TGFβR1 expression were significantly associated with chemosensitivity to DDP and tumor metastasis in NSCLC patients. Consequently, miR-181b functions as a tumor suppressor and has an important role in proliferation, chemosensitivity to DDP and metastasis of NSCLC by targeting TGFβR1/Smad signaling pathway.

Lung cancer is the leading cause of cancer morbidity and mortality worldwide. Non-small cell lung cancer (NSCLC) accounts for 80-85% of lung cancer with a 5-year survival rate of 15%^1^. Approximately two-thirds of patients are diagnosed at an advanced stage, and of the remaining patients who undergo complete surgical resection, the recurrence rate remains high (30-70%)^2^, due to the resistance to drugs and metastasis of NSCLC. Therefore, clarification of the molecular mechanisms underlying chemosensitivity and metastasis of NSCLC, establishment of an effective prognostic biomarkers and therapeutic targets for the malignancy are clinically important.

Recently, research on microRNA (miR) has proceeded at an accelerated rate. MiRs are a class of 22 nucleotide non-coding RNA molecules that negatively regulate the expression of target genes post-transcriptionally by binding to the 3′ untranslated region (3’UTR) of mRNA^3^. With the expansion of miRNA expression profiling efforts, miRNA abnormal expression patterns have been identified as unique signatures associated with diagnosis, staging, prognosis and response to treatment. In addition, miRNAs could regulate the sensitivity of drugs and directly involved in cancer metastasis by activating oncogenic pathways or suppressing tumor suppressor gene pathways or protein expression^4^,^5^,^6^,^7^. Many papers have been reported that miR-181b is one of the important determinants of the prognosis and/or chemosensitivity in several kinds of cancers, such as gastric cancer, colon cancer, pancreatic cancer, chronic lymophocytic leukemia and glioma^8^,^9^,^10^,^11^,^12^,^13^. With regard to NSCLC, it has been reported that miR-181b expression was significantly down-regulated in NSCLC tissues as compared with their normal counterparts. Low miR-181b expression may be a poor prognostic marker of NSCLC patients^14^. MiR-181b was down-regulated in A549/DDP cells compared with A549 cells and modulated multidrug resistance by targeting BCL-2^15^. Those results suggested that miR-181b might be involved in NSCLC development and chemosensitivity. However, the mechanism underlying the role of miR-181b in cell proliferation and chemosensitivity in NSCLC have not been fully elucidated. Additionally, its involvement in metastasis also remains largely unknown.

The transforming growth factor β (TGFβ) superfamily plays crucial roles in cell proliferation, apoptosis, EMT, invasion and metastasis^16^,^17^. Two types of cell-surface receptors, TGFβ receptor types 1 and 2 (TGFβR1 and TGFβR2), are mainly involved in the transmission of TGFβ signaling to perform multiple intracellular functions. Upon binding of TGFβ to its receptor TGFβR2, the active TGFβR2 recruits and causes phosphorylation of TGFβR1, which can phosphorylate the transcription factors Smad2 and Smad3, and then leads to formation of the Smad2/3/4 complex. The complex translocates into the nucleus, where the Smad transcriptional complex regulates the expression of target genes, such as C-myc, p21. Hence, TGFβR1 is the central propagator of the TGFβ signaling. Besides the canonical pathway, TGFβ has been reported to interfere with the activity of other proteins and signaling pathways, such as the PI3K/Akt pathway^18^.

In the present study, we aimed to evaluate the possible roles and related target genes of miR-181b in tumorigenesis of NSCLC. We found that miR-181b expression levels were lower in A549/DDP in comparison to A549 cells. Functional assays showed that the overexpression of miR-181b inhibited cell proliferation, chemoresistance to DDP, migration, invasion and metastasis *in vitro* and *in vivo*. Furthermore, studies identified TGFβR1 as a direct and functional target of miR-181b. TGFβR1 knockdown revealed similar effects as that of ectopic miR-181b expression, whereas overexpression of TGFβR1 attenuated the function of miR-181b in NSCLC cells. In addition, miR-181b could inactivate the TGFβR1/Smad signaling pathway. We also observed that the expression level of miR-181b and TGFβR1 were significantly correlated with chemosensitivity to DDP and metastasis in NSCLC patients. Therefore, miR-181b could regulate cell proliferation and cisplatin-chemosensitivity and metastatic capability by targeting TGFβR1/Smad signal pathway, suggesting a candidate method for NSCLC treatment.

## Results

### MiR-181b inhibits cell proliferation in NSCLC cells

**Figure 1:**
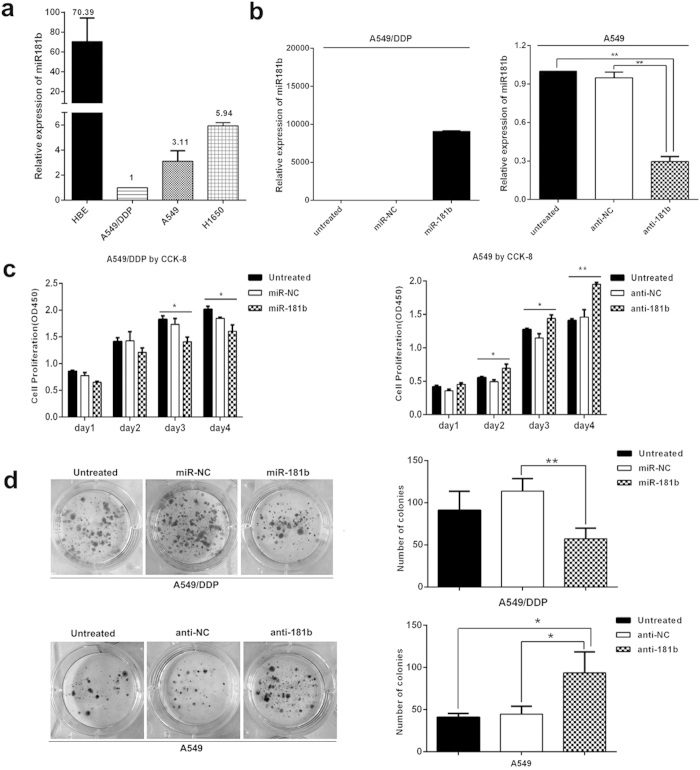
MiR-181b inhibits cell proliferation in NSCLC cells. (**a**) qPCR analysis to quantify the expression levels of miR-181b in HBE, A549/DDP, A549 and H1650 cell lines. U6 was used for normalization. (**b**) CCK analysis of cell proliferation after transfected with miR-181b mimics, miR-181b inhibitors, or control in A549/DDP or A549 cell lines. (**c**) Colony-forming efficiency was detected in A549/DDP or A549 cell lines transfected with the miR-181b mimics or miR-181b inhibitors. *P < 0.05; **P < 0.01.

In this study, we investigated the expression levels of miR-181b in HBE normal lung epithelial cell line, A549, H1650 and A549/DDP lung cancer cell lines. MiR-181b expression was significantly decreased in A549/DDP cells compared with A549 and H1650 cells, and they were lower than HBE normal lung epithelial cells, as detected by qPCR (Fig. 1a).

To study whether miR-181b could contribute to malignant phenotypes of NSCLC cells, we evaluated miR-181b on cell growth in A549/DDP, A549 and H1650 cells. qPCR assay indicated that expression level of miR-181b in A549/DDP, A549, H1650 and transfected cells (Fig. 1b and Fig. S1a). After transfection with miR-181b mimics, the proliferation of A549/DDP cells were significantly inhibited at days 3 to 4 (*P* < 0.05), whereas miR-181b inhibitor increased cell growth in A549 cells at days 2 to 4 (*P* < 0.05, Fig. 1c). Accordingly, the colony-forming efficiency of A549/DDP cells transfected with the miR-181b mimic were significantly lower than that of cells transfected with the miR-control (*P* < 0.01). Compared with anti-miR-NC transfected cell, colony-forming efficiency of in anti-miR-181b transfected A549 cells were significantly increased (*P* < 0.05, Fig. 1d). The same results were confirmed in H1650 cell (Fig. S1b,c). These results suggest that miR-181b inhibits cell proliferation in NSCLC cells.

### MiR-181b enhances chemosensitivity of NSCLC cells to DDP

**Figure 2:**
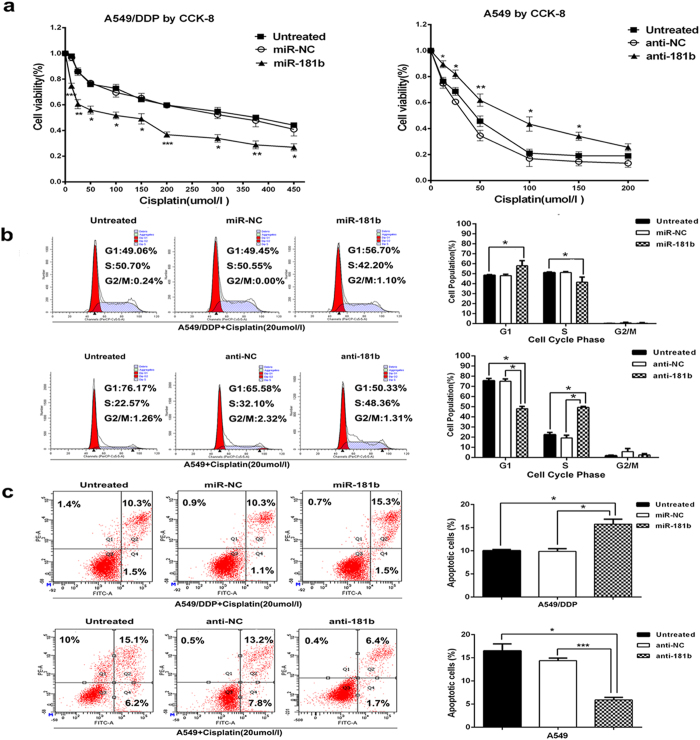
MiR-181b enhances chemosensitivity of NSCLC cells to DDP. (**a**) CCK analysis of IC50 values of DDP after transfected with miR-181b mimics, miR-181b inhibitors, or control in A549/DDP or A549 cell lines. (**b**) Flow cytometric analysis of cell cycle in A549/DDP and A549 were determined after transfected miR-181b mimics, miR-controls, miR-181b inhibitors or negative controls combined with DDP. (**c**) Flow cytometric analysis of apoptosis in A549/DDP and A549 were determined after transfected miR-181b mimics, miR controls, miR-181b inhibitors or negative controls combined with DDP. *P < 0.05; **P < 0.01; ***P < 0.001.

Next, we assessed the functional role of miR-181b in chemosensitivity of NSCLC cells to DDP. After transfected with miR-181b mimics, the IC50 of A549/DDP cells to DDP was significantly decreased (313.80 ± 17.21 umol/l *vs* 89.35 ± 3.38 umol/l, *P* < 0.01), as evidenced by the growth inhibition curve. Conversely, inhibition of miR-181b could simultaneously protected A549 and H1650 cells from cisplatin treatment (*P* <  0.001, Fig. 2a and Fig. S2a).

To determine the effects of miR-181b on the cell cycle and apoptosis of NSCLC cells when exposed to DDP treatment, flow cytometry assays were performed. After transfection with the miR-181b mimics, the percentage of cells in the G1 stage was higher than miR-NC group in A549/DDP cells (*P* < 0.05). And the percentage of cells in the S stage was lower than miR-NC (*P* < 0.05, Fig. 2b). The percent of anti-miR-181b-transfected A549 cells in G1 phase of cell cycle decreased and the percentage of cells in S phase increased compared with NC groups (*P* < 0.05, Fig. 2b). Annexin V/ PI-based apoptosis analyses revealed that transfected with miRNA-181b mimics in A549/DDP cells increases the ratio of apoptotic cells (8.13 ± 0.19% *vs* 13.50 ± 0.29%, *P* < 0.05 Fig. 2c). However, blocking miR-181b in A549 cells significantly reduced DDP-induced apoptosis (14.40 ± 0.31% *vs* 5.90 ± 0.32%, *P* < 0.001 Fig. 2c). Therefore, these data suggest that miR-181b could increase chemosensitivity of DDP by inducing cell arrest in G1 phase and apoptosis enhancement when exposed to DDP treatment.

### MiR-181b attenuates scratch, migration, invasion and modulates the epithelial-mesenchymal transition (EMT) of lung cancer cells

**Figure 3:**
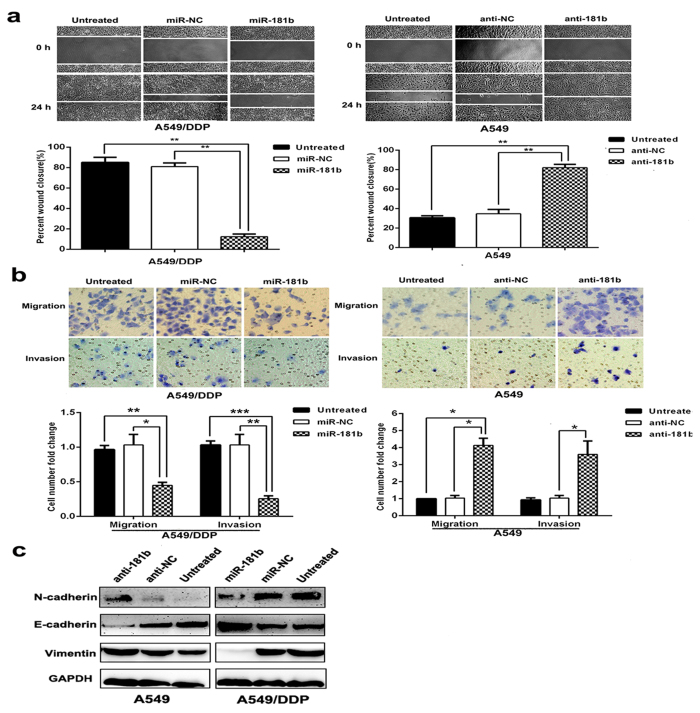
MiR-181b attenuates scratch, migration and invasion, modulates the epithelial- mesenchymal transition (EMT) of lung cancer cells. (**a**) Wound-healing assay in A549/DDP and A549 were determined after transduction with the miR-181b mimics, miR-controls, or miR-181b inhibitors or negative controls. (**b**) Transwell migration and invasion assays for A549/DDP and A549 were determined after transduction with the miR-181b mimics, miR-controls, or miR-181b inhibitors or negative controls. (**c**) Western blot detection of E-cadherin, Vimentin, N-cadherin protein expression in A549 and A549/DDP after transduction with the miR-181b inhibitors, negative controls, or miR-181b mimics or miR-controls. GAPDH was used as an internal control. *P < 0.05; **P < 0.01; ***P < 0.001.

We further studied the effects of miR-181b on cell migration and invasion using a scratch and transwell assay. A549/DDP cells transfected with miR-181b mimics showed less migratory ability than in miR-NC at 24 h after wound creation (Fig. 3a). The migratory and invasive abilities of A549/DDP cells were also reduced by 0.45 ± 0.03 and 0.26 ± 0.02 fold by over-expression of miR-181b compared with the capabilities of control cells (*P *< 0.05; *P *< 0.01; Fig. 3b). In contrast, A549 cells transfected with miR-181b inhibitors showed more migratory ability than in miR-NC at 24 h after wound creation. Migration and invasion were increased by 4.13 ± 0.24 and 3.60 ± 0.46 fold with miR-181b knockdown treatment in A549 cells (*P *< 0.05, *P *< 0.05; Fig. 3b). The same results were confirmed in H1650 cell (Fig. S3a,b).

EMT has been regarded as an important mechanism that facilitates cancer cell migration and leads to metastasis, so we also tested whether miR-181b is involved in the EMT to influence cancer metastasis. Several putative EMT-related markers, such as E-cadherin, N-cadherin and Vimentin were evaluated by Western Blotting. A549/DDP cells transfected with miR-181b mimics decreased Vimentin, N-cadherin expression and increased E-cadherin expression, and A549 cells transfected with miR-181b inhibitors showed an increase in Vimentin, N-cadherin expression and a decrease in E-cadherin expression (Fig. 3c). These observations indicate that miR-181b can also suppress the EMT of NSCLC. Collectively, our findings suggest that miR-181b negatively regulate tumor migration, invasion and EMT marker expression of NSCLC cells *in vitro*.

### TGFβR1 is a direct target of miR-181b

**Figure 4:**
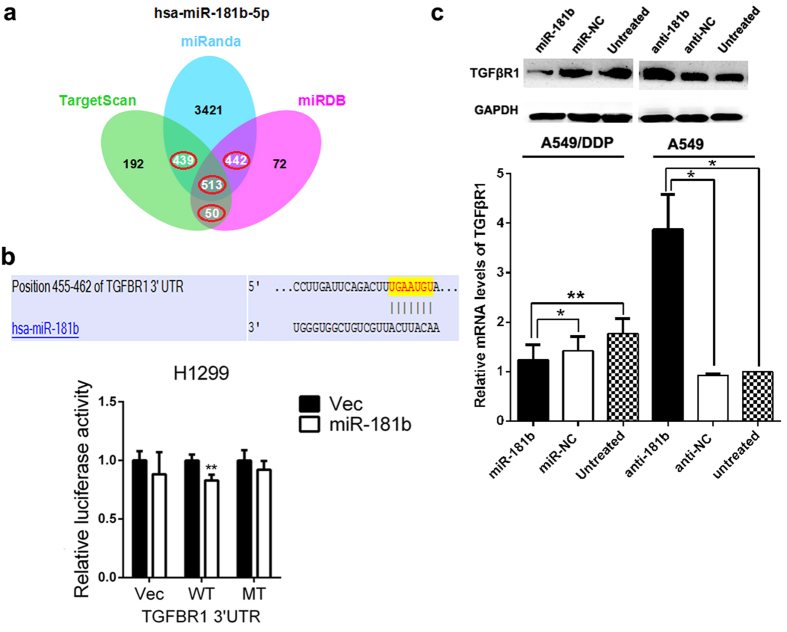
TGFβR1 is a direct target of miR-181b. (**a**) Venn diagrams showed the number of genes identified as potential targets of miR-181b using three predicting search programs of TargenSCan, miRanda and miRBD. (**b**) Relative luciferase activity was analysed after wild-type or mutant 3′-UTR reporter plasmids were co-transfected with pGLE/miR-181b or miR-181b-NC in H1299 cells. (**c**) qRT-PCR and western blot detection of TGFβR1 mRNA and protein expression in A549 or A549/DDP cells transfected with miR-181b mimics or anti-miR-181b. U6 or GAPDH was used as an internal control. *P < 0.05; **P < 0.01.

To elucidate the underlying mechanisms of the suppressive effects of miR-181b on cell proliferation, chemosensitivity to DDP and metastasis of NSCLC, we used several bioinformatics methods to help identify the target human genes of miR-181b. Among the targets predicted by the search programs of TargetScan, miRanda and miRDB, TGFβR1 was the gene that localized at the three main centers of the net comprised of 513 predicted genes (Fig. 4a).

To confirm that miR-181b can regulate TGFβR1 expression by directly binding to the TGFβR1 3′-UTR, we generated luciferase reporter constructs containing specific mutations at putative miR-181b binding site. As shown in Fig. 4b, when H1299 cells were transfected with the wild type TGFβR1 3′-UTR, co-transfection of miR-181b mimics inhibited luciferase activity (*p *= 0.002). In contrast, the effects of miR-181b mimics were eliminated in H1299 cells transfected with the mutant type TGFβR1 3′-UTR. These results suggested that miR-181b binds directly to putative TGFβR1 3′-UTR regions, as predicted.

Moreover, we found that the protein expression and mRNA levels of TGFβR1 were up-regulated in A549/DDP cells compared with A549 and H1650 cells. The expression levels of TGFβR1 decreased in A549/DDP cells transfected with miR-181b mimics and increased in A549 and H1650 cells transfected with miR-181b inhibitors (Fig. 4c and Fig. S4a).

### TGFβR1 has a critical role in miR-181b-mediated cell growth, chemosensitivity to DDP and metastasis of NSCLC cells

**Figure 5:**
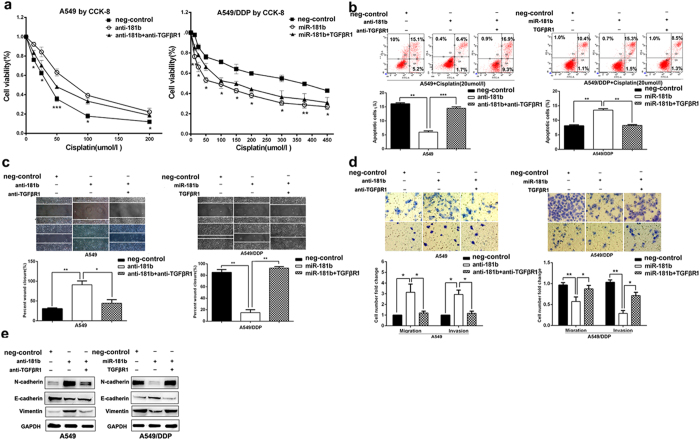
TGFβR1 has a critical role in miR-181b-mediated cell growth, chemosensitivity to DDP and metastasis of NSCLC cells. (**a**) CCK analysis of the IC50 values of DDP in A549/miR-181b inhibitors, A549/miR-181b inhibitors/si-TGFβR1, A549/DDP/miR-181b or A549/DDP/miR-181b/TGFβR1. (**b**) Flow cytometric analysis of apoptosis in A549/miR-181b inhibitors, A549/miR-181b inhibitors/si-TGFβR1, A549/DDP/miR-181b or A549/DDP/miR-181b/TGFβR1 with DDP treatment. (**c,d**) Scratch, migration, invasion assay in A549/miR-181b inhibitors, A549/miR-181b inhibitors/si-TGFβR1, A549/DDP/miR-181b or A549/DDP/miR-181b/TGFβR1. (**e**) Western blot detection of E-cadherin, Vimentin, N-cadherin protein expression in A549/miR-181b inhibitors, A549/miR-181b inhibitors/si-TGFβR1, A549/DDP/miR-181b or A549/DDP/miR-181b/ TGFβR1. GAPDH was used as an internal control. *P < 0.05; **P < 0.01; ***P < 0.001.

We further investigated whether TGFβR1 was responsible for cell growth, chemosensitivity to DDP and metastasis of NSCLC cells after regulating miR-181b expression. Through the knock-down TGFβR1 in anti-miR-181b-A549 cells, cells were more sensitive to DDP, decreased migratory, invasive capability and inhibited EMT markers than control. Through transfection of TGFβR1 in miR-181b-A549/DDP cells, cells were more resistant to cisplatin, increased migratory, invasive capability and EMT markers expression than control (Fig. 5a–e).

These results indicated that TGFβR1 has a critical role in miR-181b-mediated cell growth, chemosensitivity to DDP and metastasis of NSCLC cells.

### MiR-181b regulates a Smad-dependent and a Smad-independent TGF-β signaling by suppressing TGFβR1

**Figure 6:**
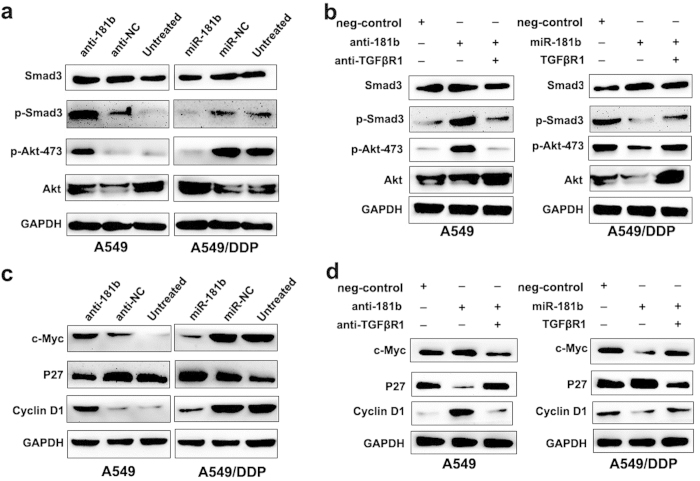
MiR-181b regulates a Smad-dependent and a Smad-independent TGF-β signaling by suppressing TGFβR1. (**a**) Western blot detect expression of Smad3, p-Smad3, p-Akt473 and Akt in A549, A549/miR-181b inhibitors, A549/DDP or A549/DDP/miR-181b. (**b**) Western blot detect expression of Smad3, p-Smad3, p-Akt473 and Akt in A549/miR-181b inhibitors, A549/miR-181b inhibitors/si-TGFβR1, A549/DDP/miR-181b or A549/DDP/miR-181b/ TGFβR1. (**c**) Western blot detect expression of c-Myc, p27 and cyclin D1 in A549, A549/miR-181b inhibitors, A549/DDP or A549/DDP/miR-181b. (**d**) Western blot detect expression of c-Myc, p27 and cyclin D1 in A549/miR-181b inhibitors, A549/miR-181b inhibitors/si-TGFβR1, A549/DDP/miR-181b or A549/DDP/miR-181b/TGFβR1. GAPDH was used as an internal control.

TGFβR1 is an important component in TGF-β signaling. Its loss or reduced expression may impair TGF-β signaling. To examine if miR-181b is involved in the abrogation of this signaling pathway, the phosphorylation of Smad3, a key protein in this pathway, was detected in A549 cells and A549/DDP cells treated with miR-181b mimics and inhibitors respectively. As shown in Fig. 6a, the introduction of miR-181b did attenuate p-Smad3 expression, whereas silencing of miR-181b increased p-Smad3 expression. Notably, the reduction of p-Smad3 expression was also observed in A549 treated with siRNA-TGFβR1, similar to the effect of miR-181b (Fig. 6b). These data indicate that miR-181b attenuates Smad-dependent TGF-β signal though suppressing TGFβR1.

PI3K/Akt signaling pathway is a central regulator in cancer cell proliferation, metastasis and EMT process. Multiple studies have suggested the existence of direct or indirect crosstalk between TGF-β signaling and PI3K/Akt signaling, so we wonder whether PI3K/Akt pathway is also involved in NSCLC aggressiveness in the presence of miR-181b-mediated TGFβR1 inactivation. To test this, we examined the expressions of p-Akt, after treating A549 and A549/DDP cells with miR-181b inhibitors and siRNA-TGFβR1 or miR-181b mimics respectively. Our results showed that miR-181b inhibitor could elevate the expressions of p-Akt in A549 cells, whereas its mimic could reduce the expressions of p-Akt in A549/DDP. As expected, siRNA-TGFβR1 displayed a consentaneous phenocopy with the effect of miR-181b mimics in A549 cells (Fig. 6a,b), suggesting the downregulated TGFβR1 could decrease PI3K/Akt pathway. Notably, miR-181b inhibitors could increase the expression of c-Myc and Cyclin D1, and reduce p27 expression in A549 cells. Conversely, miR-181b mimics could reduce the expression of c-Myc and Cyclin D1, and increase p27 expression in A549/DDP cells. Additionally, the increasing of c-Myc, Cyclin D1 expression and decreasing p27 expression were observed in A549 treated with siRNA-TGFβR1, similar to the effect of miR-181b (Fig. 6c,d).

### Expression of miR-181b in NSCLC tissues is negatively correlated with TGFβR1 expression and responses of NSCLC patients to DDP

**Figure 7:**
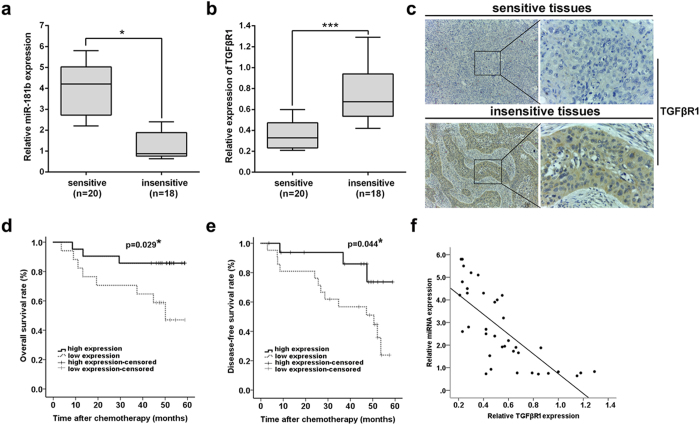
Expression of miR-181b in NSCLC tissues is negatively correlated with TGFβR1 expression and responses of NSCLC patients to DDP-based chemotherapy. (**a**) qRT-PCR detection of relative miR-181b expression in sensitive group (n* *= 20) and insensitive group (n* *= 18) NSCLC tissues. Abundance of miR-181b was normalized to U6 RNA. (**b**) qRT-PCR detection of relative TGFβR1 mRNA expression in sensitive group (n* *= 20) and insensitive group (n* *= 18) NSCLC tissues. Abundance of TGFβR1 was normalized to GAPDH. (**c**) Immunostaining of TGFβR1 protein expression in sensitive and insensitive NSCLC tissues. (**d**) The OS curves for the high-miR-181b expression group (n* *= 21) and the low-miR-181b expression group (n* *= 17). (**e**) The DFS curves for the high-miR-181b expression group (n* *= 21) and the low-miR-181b expression group (n* *= 17). (**f**) A statistically significant inverse correlation between miR-181b and TGFβR1 mRNA levels in 38 cases of NSCLC tissues.

To investigate the correlation between miR-181b/TGFβR1 dysregulation and response to DDP and prognosis of patients, the expression of miR-181b and TGFβR1 mRNA was detected in a total of 38 NSCLC patients who received surgery, primary culture and subsequent MTT assay. Based on the patient’s response to DDP, they were divided into two groups: IR > 30% as sensitive; and IR≤30% as resistant. First, qPCR was used to detect the expression of miR-181b and TGFβR1 mRNA expression, and we showed that the relative level of miR-181b expression in sensitive tissues (n* *= 20) was significantly higher than that in insensitive tissues (n* *= 18) (*P *< 0.05; Fig. 7a). However, the relative level of TGFβR1 mRNA expression in sensitive groups was significantly lower than that in insensitive groups (*P *< 0.001; Fig. 7b). Immunostaining of TGFβR1 protein expression indicated that the staining of TGFβR1 protein was stronger in the insensitive tissues than in the sensitive tissues (Fig. 7c).

The correlation of miR-181b expression with prognosis of NSCLC patients was investigated by Kaplan-Meier analysis and log-rank test. As shown in Fig. 7d,e, the status of miR-181b expression was found to be closely correlated with both overall survival (OS) and disease-free survival (DFS) of patients (*P *= 0.029 and 0.044, respectively). Furthermore, by linear regression analysis, it was found that there was an inverse correlation between relative miR-181b and TGFβR1 mRNA expression in NSCLC tissues from patients (Fig. 7f). These data suggested that miR-181b expression in tumor tissues was negatively correlated with TGFβR1expression and responses of NSCLC patients to DDP.

To further support our finding, we finally investigated the clinical relevance of miR-181b and TGFβR1 in NSCLC patients. We observed that the lower expression of miR-181b was associated with bigger tumor size, higher pT status and pTNM stage. The higher expression of TGFβR1 was correlated with greater possibility of lymph node metastasis (Supplementary Table S1), supporting that miR-181b-mediated TGFβR1 down-regulation was closely linked to NSCLC proliferation and aggressiveness. In addition, when all of the clinicopathological factors were evaluated, multivariate analysis using the Cox proportional hazard model indicated that status of miR-181b might be an independent factor for prediction of poor OS (HR: 0.022, 95% CI: 0.001-0.328; *p *= 0.006) in NSCLC patients (Supplementary Table S2).

To further validate these findings, we searched the Oncomine database for the prognostic value of TGFβR1 in NSCLC. Two data sets showed that the high levels of TGFβR1 are significantly correlated with short OS in NSCLC patients. Two data sets confirmed that TGFβR1 are significantly higher expression in metastasis tissue compared with primary site (Supplementary Table S3).

Collectively, these data suggest that the miR-181b/TGFβR1 plays an important role in chemosensitivity and metastasis of NSCLC.

### MiR-181b regulates chemosensitivity to DDP and metastasis *in vivo*

**Figure 8:**
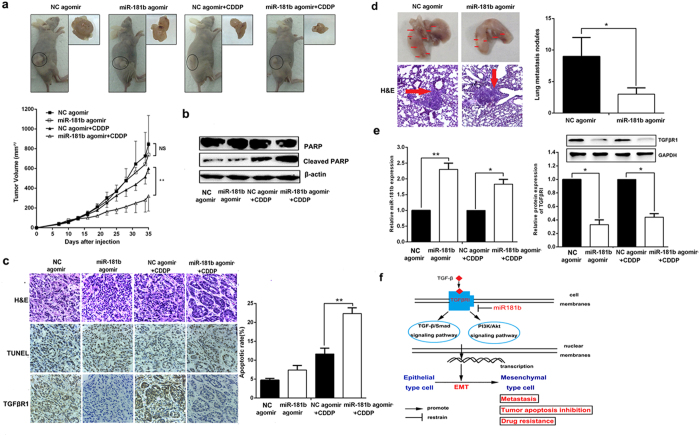
MiR-181b regulates chemosensitivity to DDP and metastasis *in vivo*. Mice were treated with DDP (3.0 mg kg^−^1 body weight; i.p., thrice) or with 0.1 ml PBS (pH 7.4; i.p., thrice). (**a**) Representative images of tumor growth 28 days after injection using miR-181b agomir or miR-181b-NC agomir. Representative images of tumor volume growth curves (left). (**b**) Western blotting detect the expression of PARP and cleaved PARP in tumors developed from miR-181b agomir or miR-181b-NC agomir treated. (**c**) Immunostaining of TGFβR1 protein expression in tumors developed from A549/DDP/ miR-181b agomir or A549/DDP/ miR-NC agomir cells treated with DDP or PBS. Up: H&E staining; middle: TUNEL assay; down: immunostaining. Original magnification: × 200. The bar graph is the apoptosis rate calculated of TUNEL assay. (**d**) Representative photos of mouse lungs and images of the histological inspection of mouse lungs for the presence of microscopic lesions at 7 weeks after tail vein injection with A549/DDP cells and injected subcutaneously miR-181b agomir or miR-181b-NC agomir. (**e**) qPCR and Western blotting detect the expression of miR-181b , TGFβR1 in tumors developed from miR-181b agomir or miR-181b-NC agomir treated. Data were presented as mean ± s. d., (**f**) Hypothesized mechanisms of miR-181b regulate cisplatin-resistant and metastasis in NSCLC. *P < 0.05; **P < 0.01.

To explore whether miR-181b could influence lung cancer chemosensitivity to DDP *in vivo*, s.c. tumors were developed in nude mice followed by treatment with agomir-181b or agomir-NC. All Nude mice were treated with DDP. As shown in Fig. 8a, the tumors treated with agomir-181b grew significantly slower than those with agomir-NC after the treatment with DDP. At 35 days after inoculation, the average tumor volume of A549/DDP/agomir-181b groups was significantly lower than that of A549/DDP/agomir-NC following DDP treatment (*P *< 0.01). Agomir-181b with DDP increased the expression of cleaved PARP compared to NC-agomir with DDP (Fig. 8b). TUNEL assay showed that the apoptotic rate of tumors developed from A549/DDP/agomir-181b groups (22.33 ± 0.88%) was significantly higher than that of tumors developed from A549/DDP/agomir-NC groups (11.67 ± 0.88%) following DDP treatment (*P *< 0.01; Fig. 8b,c).

Thus, we further investigated whether miR-181b could affect NSCLC cell metastasis *in vivo*. A549/DDP cells were injected into the lateral tail veins of nude mice and established a model to imitate metastasis formation (n* *= 6). As expected, miR-181b suppressed lung metastasis formation. The number of lung metastasis nodules was significantly decreased in the agomir-181b group and increased in the agomir-NC group (*P *< 0.05, Fig. 8d). Taken together, these results suggest that miR-181b is negative regulators of NSCLC metastasis.

To clarify the cellular mechanisms underlying miR-181b-mediated tumor chemosensitivity to DDP and metastasis, resected tissues from those treated xenograft tumors were analyzed to verify miR-181b and TGFβR1 expression. As shown in Fig. 8c,e, miR-181b could significantly decrease the expression of TGFβR1 *in vivo*.

All these data indicated that treated with miR-181b could rescue chemosensitivity to DDP and reduce metastasis in NSCLC cells vivo.

## Discussion

Increasing evidence suggests that deregulation of miRNA are detected in many human cancers, including NSCLC, demonstrating various functions by interaction with tumor-related genes^19^,^20^. These miRNAs have regulatory roles in the pathogenesis of cancer in humans, through the suppression of genes involved in cell proliferation, differentiation, apoptosis, resistance and metastasis^21^,^22^,^23^. Therefore, identification of tumor-related miRNA and their direct target genes is critical for understanding the biological significance of miRNA in tumorigenesis and may reveal novel therapeutic targets for the effective treatment of patients. Among these, miR-181b was identified as one of the most important miRNAs contributing to tumor initiation and progression.

Previous reports showed that miR-181b may function as either an oncogene or a tumor suppressor in different cancer types. For example, miR-181b promotes cell proliferation and inhibits apoptosis in ovarian cancer and cervical cancer^24^,^25^. MiR-181b as an oncogenic microRNA enables monitoring of early breast cancer in serum^26^. In contrast, miR-181b inhibits cell growth in gastric cancer by targeting CREB1^27^ and increases drug sensitivity in acute myeloid leukemia via targeting HMGB1 and Mcl-1^28^. MiR-181b modulates glioma cell sensitivity to temozolomide by targeting MEK1^13^. In our study, we found that miR-181b inhibited the proliferation of NSCLC *in vitro*. MiR-181b enhanced chemosensitivity of NSCLC cells to DDP *in vitro* and *in vivo*. These results suggested that miR-181b was a tumor suppressor in NSCLC.

Recently, miR-181b was reported to have suppressive effects on tumor metastasis. MiR-181b downregulated NOVA1 to suppress migration and invasion in astrocytoma^29^. In this study, the potential involvement of miR-181b in NSCLC metastasis was investigated. The ectopic expression of miR-181b markedly suppressed the migratory and invasive capacities of NSCLC cells *in vitro*. MiR-181b agomir significantly reduced the lung metastases of A549/DDP *in vivo*. Furthermore, miR-181b downregulation was also found to involve in NSCLC patients with metastasis. These results suggested that miR-181b was a negative regulator in NSCLC invasion and metastasis.

TGFβR1 was further identified as a new direct and functional target of miR-181b using several different miRNA target prediction programs and experimental validation. We demonstrated that miR-181b binds to the 3′-UTR of TGFβR1 by using dual-luciferase reporter assays. In addition, miR-181b can inhibit TGFβR1 mRNA and protein expression levels. It has been demonstrated that TGFβ signaling has two distinct and opposite roles in cancer progression and metastasis. During early stages of carcinogenesis, TGFβ strongly suppresses cell growth and acts as a tumor suppressor^30^,^31^,^32^,^33^. In contrast, in late stages of cancer progression, it promotes tumor progression, EMT, invasion and metastasis^34^,^35^. This dramatic change in TGFβ function reflects a variety of dynamic alterations that occur within tumor cells.

However, the mechanism underlying the role of TGFβR1 in NSCLC also remains largely unclear. Some research reported that TGFβR1 was down regulated in NSCLC^36^,^37^. Lei Z *et al*. found that MiR-142-3p repressed TGF-β-induced growth inhibition through repression of TGFβR1 in non-small cell lung cancer^38^. While, Hou and Okayama Lung Statistics shown that TGFβR1 was upregulation in lung adenocarcinoma who was dead at 3 or 5 year compared to alive at 3 or 5 year in Oncomine database. Bhattacharjee Lung Statistics shown that TGFβR1 was upregulated 2.05 fold in metastasis than primary site in lung cancer. In this study, we observed that TGFβR1 knock-down reduced cell proliferative and invasive ability, similar effects as that of ectopic miR-181b expression. The relative level of TGFβR1 mRNA expression in insensitive tumor tissues was significantly higher than that in sensitive tumor tissues. These results suggested that TGFβR1 has the oncogenic property in NSCLC. It was found that there was an inverse correlation between relative miR-181b and TGFβR1 mRNA expression in NSCLC tissues. Furthermore, several small molecular weight compounds and antibodies inhibiting the TGFβ signaling pathway are now under clinical trials. LY-573636, the TGFβR1 inhibitor, is in phase II clinical trials for metastatic breast cancer, metastatic non-small-cell lung cancer, and malignant melanoma^39^. SB-431542, the most widely used TGFβR1 inhibitor, has been shown to inhibit tumor metastasis in breast cancer^40^, glioma^41^, and renal cell carcinoma^42^ in the preclinical stage. YR-290, the TGFβR1 inhibitor, also can inhibit the kinase activity of TGFβR1 and TGFβ-mediated metastasis in breast cancer^43^. Therefore, it has been proposed that MiR-181b/TGFβR1 may have potential clinical use in the future.

It is also known that TGF-β and PI3K/AKT signal transductions are two pivotal pathways that control cell function. Importantly, PI3K/Akt pathway could be directly or indirectly regulated by TGF-β^44^,^45^,^46^. In the present study, we provided further evidence that miR-181b-mediated TGFβR1 inactivation not only pulled down the TGF-β signaling but also inactivated PI3K/Akt pathway in NSCLC. Bresin A *et al*. also found that the expression of pAkt was influenced by miR-181b in chronic lymphocytic leukemia^47^. Subsequently, we observed the obvious expression alterations of some important genes, c-Myc, Cyclin D1 and p27, related to cell proliferation and cell cycle, in NSCLC cells in the presence of miR-181b-mediated TGFβR1 inactivation. In particular, the knockdown of TGFβR1 by siRNA displays a consentaneous phenomenon with the effect of miR-181b. These date support a major involvement of miR-181b-medited TGFβR1 inactivation that drives down the TGF-β signaling and suppresses PI3K/Akt pathway in NSCLC proliferation and aggressiveness.

In conclusion, we demonstrated that miR-181b inhibited cell proliferation, augmented the chemosensitivity to DDP, suppressed migration and invasion in NSCLC cells *in vitro* and *in vivo*. Furthermore, MiR-181b may increase chemosensitivity to DDP and suppress the invasion and metastasis of NSCLC cells through directly targeting the TGFβR1 signaling pathway. MiR-181b and TGFβR1 were negative correlated, and associated with chemosensitivity to DDP and metastasis in NSCLC patients. Thus, miR-181b plays multiple tumor suppressive roles in NSCLC cells. Our findings suggest that miR-181b/TGFβR1 might be a promising prognostic and therapeutic target to increase chemosensitivity to DDP and suppress metastasis in NSCLC.

## Methods

### Cell lines and culturing

The HBE normal lung epithelial cell line, human lung cancer cell line A549, A549/DDP and H1650 were obtained from Heilongjiang Cancer Institute (Harbin, China). Cells were cultured in RPMI-1640 medium supplemented with 10% fetal bovine serum and 50 U/ml penicillin/50 mg/ml streptomycin, then incubated at 37°C in a humidified atmosphere of 5% CO_2_. To maintain the MDR phenotype, DDP (1 ug/ml) was added to the culture media for A549/DDP cells.

### Real time-PCR

Total cellular RNA from the cultured cells was isolated using Trizol reagent (Invitrogen). RNA samples (500 ng each) were then reverse-transcribed into cDNA with miRNA-181b reverse transcriptase primers using a TaqMan MicroRNA Reverse Transcription kit (Applied Biosystems). Levels of miRNA-181b and U6 expression were determined by qPCR with TaqMan MicroRNA Assays and an ABI 7500 machine (Applied Biosystems). The levels of mature miRNA-181b expression were then normalized to U6 and calculated as the inverse log of the ΔCt (Relative mRNA abundance was calculated as 2^–ΔCt [ΔCt^ **=  ^Ct (miR–181b) – Ct(U6)]^). All procedures were performed following the manufacturer’s instructions.

### Western blot analysis

The cells or tissues lysed in lysis buffer. 50 μg of protein sample each was separated by SDS-PAGE (10% gels) and transferred onto a 0.45 μm polyvinylidene fluoride (PVDF) membrane. The proteins were probed with TGFRβR1, E-cadherin, N-cadherin, Vimentin, p-Smad3, Smad3, p-Akt, c-Myc, p27, cyclin D1, PARP and cleaved PARP antibody (Cell Signaling). The bound antibodies were detected using ECL Western Blotting Detection system. Equivalence of protein loading was assessed using (mouse anti-β-actin or GAPDH antibody, Sigma).

### MiRNA mimics and inhibitor, gene transfection

The cells were cultured in 6-well plates to 40% confluence. MiR-181b mimics, inhibitor, negative control TGFβR1 or si-TGFβR1 (Sigma) were mixed with Lipofectamine 2000 (Invitrogen), and then added to the cell culture medium according to the manufacturer’s instructions. After 24 h of transfection, total RNA and protein were prepared from the cells and subjected to qRT-PCR and western blot analyses, respectively.

### Dual luciferase reporter assay

1 × 10^5^ H1299/well were cultured in 24-well plates, transfected with TGFβR1-3′UTR- wt or TGFβR1-3′UTR-mt and mi-181b or mi-NC using Lipofectamine 2000 (Invitrogen, USA) according to the manufacturer’s protocol as described. Luciferase activity was measured 24 h after transfection using the Dual Luciferase Reporter Assay System (Promega) and normalized to Renilla luciferase activity.

### Cell proliferation assay and primary culture of cells from NSCLC patients

1 × 10^5^/well the suspensions of NSCLC cell lines were incubated in 96-well plates and grew at 37°C with different concentrations of cisplatin (0~200 umol/l for A549 cells; 0~450 umol/l for A549/DDP cells). Each patient had signed informed consent for medical record review and tissue sample donation. This study was approved by the institutional review board at Harbin Medical University and conducted according to all current ethical guidelines. Each fresh specimen from NSCLC patients was homogenized and digested in 0.25% trypsin. The dispersed cancer cells were filtered through an 80 mm nylon mesh, washed twice in 500 mL RNase free PBS, centrifuged at 1000 rpm for 5 min, and then incubated in RPMI 1640 medium supplemented with 10% fetal bovine serum and 5% penicillin/streptomycin at 37°C in 5% CO2. The NSCLC cells from each patient were seeded at a density of 1 × 10^5^ per well in 96-well plates with culture medium for 24 h. Then the cells were treated with cisplatin. After treatments, the medium were removed and added RPMI1640 of 90 mL and CCK-8 of 10 mL. The cells on the plate were incubated for 3 h in the incubator. The absorbance at 450 nm wavelength was measured on an automated reader. The inhibition rate (IR) was calculated.

IR * *=  (OD_control group_ – OD_experimental group_)/OD_control group_ ×100%. IR > 30% was defined as sensitive and IR ≤30% as resistant. The data were presented as mean ± standard deviation (SD) from four replicate wells per microtiter plate^48^,^49^,^50^.

### Colony formation assay

After 24 h post-transfection, the cells were diluted and replaced in six-well plates. After 10 days, visible colonies were fixed with methanol, stained with crystal violet, counted and normalized to the control group. The experiments were performed at least three times.

### Flow cytometry

Cells were incubated with culture medium containing DDP for 24 h after transfection with miR-181b mimics, inhibitor or negative control. For apoptosis analysis, cell were then collected, washed with PBS, resuspended in 100 μl of 1X binding buffer and stained with 5 μl Annexin V and 5 μl of PI (Becton-Dickinson) at room temperature for 15 min in the dark. For cell cycle analysis, cells were washed with ice-cold PBS and fixed with 70% ethanol overnight at –20 °C. Fixed cells were rehydrated in PBS for 10 min and subjected to PI/RNase staining. A flow cytometer (Becton-Dickinson) was utilized to evaluate the apoptotic levels and cell cycle in each sample following the manufacturer’s instructions.

### Wound-healing assay

Cells were seeded in six-well plates and incubated to generate confluent cultures. Wounds were scratched in the cell monolayer using a 200 ul sterile pipette tip. The cells were rinsed with phosphate- buffered saline. The migration of the cells at the edge of the scratch was monitored at time 0 and 24 h, respectively. The cells were stained and photographed.

### Cell migration and invasion assay

After transfected, cells were plated in medium without serum in the top chamber of a transwell (Corning). The bottom chamber contained standard medium with 10% fetal bovine serum. After 24 h incubation, the cells that had migrated to the lower surface of the membrane were fixed with 90% ethanol, stained with crystal violet and photographed under microscope. Cell numbers were counted under a light microscope at × 400 magnification.

### Immunohistochemistry (IHC)

Each patient had signed informed consent for medical record review and tissue sample donation. This study was approved by the institutional review board at Harbin Medical University and conducted according to all current ethical guidelines. Tissue sections were immersed in MEDTA, bathed in a steamer at 100°C for 15 min and incubated in methanol containing 0.3%H_2_O_2_ for 15 min. The slides were incubated with for TGFβR1 primary antibody (1:800 dilution, Abcam), stained using DAB and counterstained using hematoxylin. The staining results were interpreted independently and blindly by two pathologists. For each slide, three to five randomly selected fields were evaluated. For each field, the percentage of DAB-positive- tumor cells was calculated as: [(number of DAB-positive tumor cells/total number of tumor cells) ×100]. The relative staining intensity was defined as negative for 5%; weak (+) for 5-25%; moderate (++) for 25-50%, and strong (+++) for 50% of the tumor cells stained positive for TGFβR1.

### *In vivo* chemosensitivity and metastasis assay

All animal experiments were undertaken in accordance with the NIH Guide for the Care and Use of Laboratory Animals, with the approval of the Scientific Investigation Board of the affiliated tumor hospital of Harbin Medical University. BALB/c athymic nude mice (male, 4–6 weeks old) were purchased from the Shanghai Experimental Animal Center of Chinese Academy of Sciences (Shanghai, P.R. China) and bred at pathogen-free conditions in Animal Center of the affiliated tumor hospital of Harbin Medical University. 4 × 10^6^ A549/DDP cells were injected subcutaneously into the left flank of each mice. After 8 days, the transplanted nude mice were randomly divided into three groups (n* *= 6 each). Agomir-miR-181b or agomir-NC (RiboBio Co., Ltd, Guangzhou, China) was directly injected into the implanted tumor at the dose of 1 nmol per mouse every 4 days for seven times. Tumor volume (V) was monitored by measuring the length (L) and width (W) and calculated with the formula V* *=  (L ×W^2^) × 0.5. Upon termination, each mouse was weighed and tumors were harvested for immunohistochemistry analysis, Western blot analysis and qPCR. The immunohistochemistry analysis was performed and measured according to our previously described method.

For tumor metastasis analysis, nude mice were inoculated with 1 × 10^6^ viable A549/DDP cells in 200 ml of phosphate buffered saline via tail vein injection. Agomir-miR-181b or agomir-NC was injected subcutaneously into the right upper flank region of mice at the dose of 1 nmol per mouse every 4 days for seven times. Four or seven weeks later, mice were sacrificed, and the lungs were harvested and fixed in formalin. The fixed samples were then embedded in paraffin, the section were stained with H&E and analyzed for presence of metastases.

### Statistical analysis

The experiments were repeated a minimum of three times. Statistical analysis was performed using SPSS 18.0. The results are expressed as the means ± SD. A one-way ANOVA test, or two-tailed Student t test, was used to compare continuous variables. *P *< 0.05 was considered to be statistically significant.

## Additional Information

**How to cite this article**: Wang, X. *et al*. MiR-181b regulates cisplatin chemosensitivity and metastasis by targeting TGFβR1/Smad signaling pathway in NSCLC. *Sci. Rep*. **5**, 17618; doi: 10.1038/srep17618 (2015).

## Supplementary Material

Supplementary Information

## References

[b1] Siegel R., Naishadham D. & Jemal A. Cancer statistics, 2013. CA Cancer J Clin 63, 11–30, doi: 10.3322/caac.21166 (2013). 23335087

[b2] Keith R. L. & Miller Y. E. Lung cancer chemoprevention: current status and future prospects. Nat Rev Clin Oncol 10, 334–343, doi: 10.1038/nrclinonc.2013.64 (2013). 23689750 PMC4687734

[b3] Bartel D. P. MicroRNAs: genomics, biogenesis, mechanism, and function. Cell 116, 281–297 (2004). 14744438 10.1016/s0092-8674(04)00045-5

[b4] Chen Y., Gelfond J., McManus L. M. & Shireman P. K. Temporal microRNA expression during *in vitro* myogenic progenitor cell proliferation and differentiation: regulation of proliferation by miR-682. Physiol Genomics 43, 621–630, doi: 10.1152/physiolgenomics. 00136.2010 (2011). 20841498 PMC3110887

[b5] Pallante P. . MicroRNA deregulation in human thyroid papillary carcinomas. Endocr Relat Cancer 13, 497–508, doi: 10.1677/erc.1.01209 (2006). 16728577

[b6] Schetter A. J. . MicroRNA expression profiles associated with prognosis and therapeutic outcome in colon adenocarcinoma. JAMA 299, 425–436, doi: 10.1001/jama.299.4.425 (2008). 18230780 PMC2614237

[b7] Zhai H. & Ju J. Implications of microRNAs in colorectal cancer development, diagnosis, prognosis, and therapeutics. Front Genet 2, doi: 10.3389/fgene.2011.00078 (2011). PMC3221387 22114584

[b8] Jiang J. . Prognostic significance of miR-181b and miR-21 in gastric cancer patients treated with S-1/Oxaliplatin or Doxifluridine/Oxaliplatin. PLoS One 6, e23271, doi: 10.1371/journal.pone.0023271 (2011). 21876743 PMC3158077

[b9] Liu J., Shi W., Wu C., Ju J. & Jiang J. miR-181b as a key regulator of the oncogenic process and its clinical implications in cancer (Review). Biomed Rep 2, 7–11, doi: 10.3892/br.2013.199 (2014). 24649060 PMC3917097

[b10] Nakajima G. . Non-coding MicroRNAs hsa-let-7g and hsa-miR-181b are Associated with Chemoresponse to S-1 in Colon Cancer. Cancer Genomics Proteomics 3, 317–324 (2006). 18172508 PMC2170889

[b11] Takiuchi D. . Involvement of microRNA-181b in the gemcitabine resistance of pancreatic cancer cells. Pancreatology 13, 517–523, doi: 10.1016/j.pan.2013.06.007 (2013). 24075517

[b12] Visone R. . miR-181b is a biomarker of disease progression in chronic lymphocytic leukemia. Blood 118, 3072–3079, doi: 10.1182/blood-2011-01-333484 (2011). 21636858 PMC3175784

[b13] Wang J., Sai K., Chen F. R. & Chen Z. P. miR-181b modulates glioma cell sensitivity to temozolomide by targeting MEK1. Cancer Chemother Pharmacol 72, 147–158, doi: 10.1007/s00280-013-2180-3 (2013). 23645289

[b14] Yang J., Liu H., Wang H. & Sun Y. Down-regulation of microRNA-181b is a potential prognostic marker of non-small cell lung cancer. Pathol Res Pract 209, 490–494, doi: 10.1016/j.prp.2013.04.018 (2013). 23827213

[b15] Zhu W., Shan X., Wang T., Shu Y. & Liu P. miR-181b modulates multidrug resistance by targeting BCL2 in human cancer cell lines. Int J Cancer 127, 2520–2529, doi: 10.1002/ijc.25260 (2010). 20162574

[b16] Massague J., Seoane J. & Wotton D. Smad transcription factors. Genes Dev 19, 2783–2810, doi: 10.1101/gad.1350705 (2005). 16322555

[b17] Schmierer B. & Hill C. S. TGFbeta-SMAD signal transduction: molecular specificity and functional flexibility. Nat Rev Mol Cell Biol 8, 970–982, doi: 10.1038/nrm2297 (2007). 18000526

[b18] Bierie B. & Moses H. L. Tumour microenvironment: TGFbeta: the molecular Jekyll and Hyde of cancer. Nat Rev Cancer 6, 506–520, doi: 10.1038/nrc1926 (2006). 16794634

[b19] Almeida M. I., Reis R. M. & Calin G. A. MicroRNA history: discovery, recent applications, and next frontiers. Mutat Res 717, 1–8, doi: 10.1016/j.mrfmmm.2011.03.009 (2011). 21458467

[b20] Boeri M., Pastorino U. & Sozzi G. Role of microRNAs in lung cancer: microRNA signatures in cancer prognosis. Cancer J 18, 268–274, doi: 10.1097/PPO.0b013e318258b743 (2012). 22647364

[b21] Ahmad J. . MicroRNA in carcinogenesis & cancer diagnostics: a new paradigm. Indian J Med Res 137, 680–694 (2013). 23703335 PMC3724248

[b22] Besse A., Sana J., Fadrus P. & Slaby O. MicroRNAs involved in chemo- and radioresistance of high-grade gliomas. Tumour Biol 34, 1969–1978, doi: 10.1007/s13277-013-0772-5 (2013). 23568705

[b23] Tang J., Ahmad A. & Sarkar F. H. The role of microRNAs in breast cancer migration, invasion and metastasis. Int J Mol Sci 13, 13414–13437, doi: 10.3390/ijms131013414 (2012). 23202960 PMC3497334

[b24] Xia Y. & Gao Y. MicroRNA-181b promotes ovarian cancer cell growth and invasion by targeting LATS2. Biochem Biophys Res Commun 447, 446–451, doi: 10.1016/j.bbrc.2014.04. 027 (2014). 24735543

[b25] Yang L. . miR-181b promotes cell proliferation and reduces apoptosis by repressing the expression of adenylyl cyclase 9 (AC9) in cervical cancer cells. FEBS Lett 588, 124–130, doi: 10.1016/j.febslet.2013.11.019 (2014). 24269684

[b26] Sochor M. . Oncogenic microRNAs: miR-155, miR-19a, miR-181b, and miR-24 enable monitoring of early breast cancer in serum. BMC Cancer 14, 448, doi: 10.1186/1471-2407-14-448 (2014). 24938880 PMC4075993

[b27] Chen L. . MicroRNA-181b targets cAMP responsive element binding protein 1 in gastric adenocarcinomas. IUBMB Life 64, 628–635, doi: 10.1002/iub.1030 (2012). 22539488

[b28] Lu F. . miR-181b increases drug sensitivity in acute myeloid leukemia via targeting HMGB1 and Mcl-1. Int J Oncol 45, 383–392, doi: 10.3892/ijo.2014.2390 (2014). 24756163

[b29] Zhi F. . MiR-181b-5p downregulates NOVA1 to suppress proliferation, migration and invasion and promote apoptosis in astrocytoma. PLoS One 9, e109124, doi: 10.1371/journal.pone.0109124 (2014). 25299073 PMC4192361

[b30] Ikushima H. & Miyazono K. TGFbeta signalling: a complex web in cancer progression. Nat Rev Cancer 10, 415–424, doi: 10.1038/nrc2853 (2010). 20495575

[b31] Itoh S. & Itoh F. Implication of TGF-beta as a survival factor during tumour development. J Biochem 151, 559–562, doi: 10.1093/jb/mvs042 (2012). 22528667

[b32] Roberts A. B. & Wakefield L. M. The two faces of transforming growth factor beta in carcinogenesis. Proc Natl Acad Sci USA 100, 8621–8623, doi: 10.1073/pnas.1633291100 (2003). 12861075 PMC166359

[b33] Wakefield L. M. & Roberts A. B. TGF-beta signaling: positive and negative effects on tumorigenesis. Curr Opin Genet Dev 12, 22–29 (2002). 11790550 10.1016/s0959-437x(01)00259-3

[b34] Papageorgis P. TGFbeta Signaling in Tumor Initiation, Epithelial-to-Mesenchymal Transition, and Metastasis. J Oncol 2015, 587193, doi: 10.1155/2015/587193 (2015). 25883652 PMC4389829

[b35] Tang B. . TGF-beta switches from tumor suppressor to prometastatic factor in a model of breast cancer progression. J Clin Invest 112, 1116–1124, doi: 10.1172/jci18899 (2003). 14523048 PMC198530

[b36] Sun J. . A haplotype of TGFBR1 is predominantly found in non-small cell lung cancer patients displaying TGFBR1 allelic-specific expression. Oncol Rep 25, 685–691, doi: 10.3892 /or.2011.1135 (2011). 21225232 10.3892/or.2011.1135

[b37] Zhao J. . Infrequently methylated event at sites –362 to –142 in the promoter of TGF beta R1 gene in non-small cell lung cancer. J Cancer Res Clin Oncol 134, 919–925, doi: 10.1007/ s00432-008-0392-4 (2008). 18421475 10.1007/s00432-008-0392-4 PMC12160756

[b38] Lei Z. . MiR-142-3p represses TGF-beta-induced growth inhibition through repression of TGFbetaR1 in non-small cell lung cancer. FASEB J 28, 2696–2704, doi: 10.1096/fj.13-247288 (2014). 24558198

[b39] Nagaraj N. S. & Datta P. K. Targeting the transforming growth factor-beta signaling pathway in human cancer. Expert Opin Investig Drugs 19, 77–91, doi: 10.1517/13543780903382609 (2010). PMC2796203 20001556

[b40] Liu I. M. . TyGFbeta-stimulated Smad1/5 phosphorylation requires the ALK5 L45 loop and mediates the pro-migratory TGFbeta switch. EMBO J 28, 88–98, doi: 10.1038/emboj.2008.266 (2009). 19096363 PMC2634733

[b41] Hjelmeland M. D. . SB-431542, a small molecule transforming growth factor-beta- receptor antagonist, inhibits human glioma cell line proliferation and motility. Mol Cancer Ther 3, 737–745 (2004). 15210860

[b42] Sjolund J. . The notch and TGF-beta signaling pathways contribute to the aggressiveness of clear cell renal cell carcinoma. PLoS One 6, e23057, doi: 10.1371/journal.pone.0023057 (2011). 21826227 PMC3149633

[b43] Fang Y. . Inhibition of breast cancer metastases by a novel inhibitor of TGFbeta receptor 1. J Natl Cancer Inst 105, 47–58, doi: 10.1093/jnci/djs485 (2013). 23178439

[b44] Kato M. . TGF-beta activates Akt kinase through a microRNA-dependent amplifying circuit targeting PTEN. Nat Cell Biol 11, 881–889, doi: 10.1038/ncb1897 (2009). 19543271 PMC2744130

[b45] Yi J. Y., Shin I. & Arteaga C. L. Type I transforming growth factor beta receptor binds to and activates phosphatidylinositol 3-kinase. J Biol Chem 280, 10870–10876, doi: 10.1074/jbc.M413223200 (2005). 15657037

[b46] Zhang L., Zhou F. & ten Dijke P. Signaling interplay between transforming growth factor-beta receptor and PI3K/AKT pathways in cancer. Trends Biochem Sci 38, 612–620, doi: 10.1016/j.tibs.2013.10.001 (2013). 24239264

[b47] Bresin A. . miR-181b as a therapeutic agent for chronic lymphocytic leukemia in the Emicro-TCL1 mouse model. Oncotarget 6, 19807–19818 (2015). 26090867 10.18632/oncotarget.4415 PMC4637322

[b48] Chen Z. . Screen anticancer drug *in vitro* using resonance light scattering technique. Talanta 77, 1365–1369, doi: 10.1016/j.talanta.2008.09.016 (2009). 19084650

[b49] Kuang Y. H. . RNA interference targeting the CD147 induces apoptosis of multi-drug resistant cancer cells related to XIAP depletion. Cancer Lett 276, 189–195, doi: 10.1016/j.canlet.2008.11.010 (2009). 19097686

[b50] Zhao Y. . ABCC3 as a marker for multidrug resistance in non-small cell lung cancer. Sci Rep 3, 3120, doi: 10.1038/srep03120 (2013). 24176985 PMC3814586

