# Author Correction: MiR-181b regulates cisplatin chemosensitivity and metastasis by targeting TGFβR1/Smad signaling pathway in NSCLC

**DOI:** 10.1038/s41598-022-25595-3

**Published:** 2022-12-15

**Authors:** Xiaoyuan Wang, Xuesong Chen, Qingwei Meng, Hu Jing, Hailing Lu, Yanmei Yang, Li Cai, Yanbin Zhao

**Affiliations:** 1 Department of Internal Medical Oncology, Harbin Medical University Cancer Hospital, Harbin, Heilongjiang Province China; 2 Cancer Research Institute, Harbin Medical University, Harbin, Heilongjiang Province China

Correction to: *Scientific Reports* https://doi.org/10.1038/srep17618, published online 01 December 2015

This Article contains errors in Figure 2C and Figure S1C.

In Figure 2, the image representing the “miR-NC” group in section “A549/DDP+Cisplatin (20 umol/l)” is swapped with the image representing the “Untreated” group in section “A549/DDP+Cisplatin (20 umol/l)”.

Furthermore, the image representing the “Untreated - Q1” group in section “A549/DDP+Cisplatin (20 umol/l)” incorrectly captions the percentage of the data as 0.9% instead of 1.0%; the image representing the “Untreated - Q2” group in section “A549/DDP+Cisplatin (20 umol/l)” incorrectly captions the percentage of the data as 10.3% instead of 10.4%; whereas the image representing the “Untreated - Q4” group in section “A549+Cisplatin (20 umol/l)” incorrectly captions the percentage of the data as 6.2% instead of 5.2%.

The correct Figure 2 and accompanying legend appear below.

**Figure 2:**
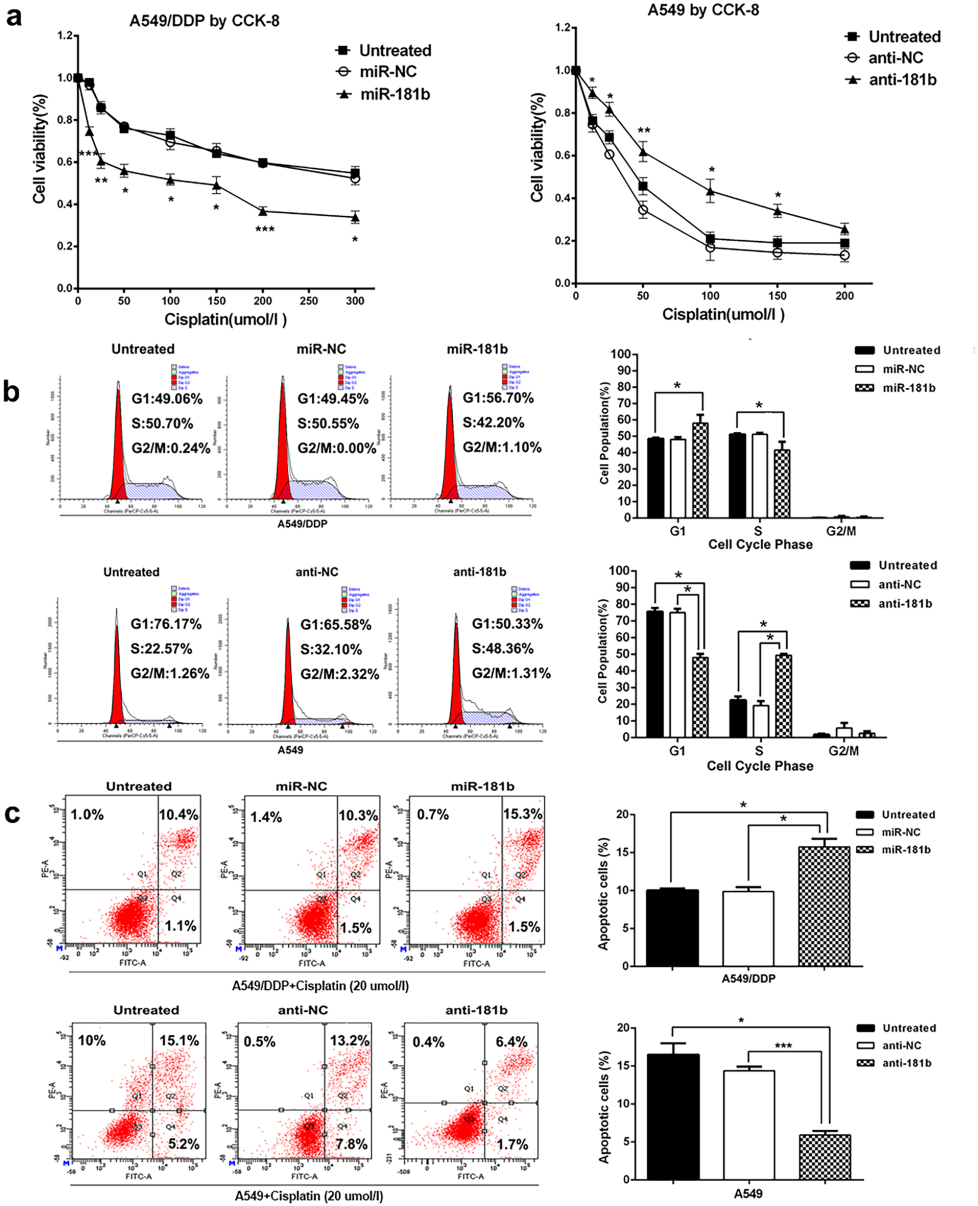
MiR-181b enhances chemosensitivity of NSCLC cells to DDP. (**a**) CCK analysis of IC50 values of DDP after transfected with miR-181b mimics, miR-181b inhibitors, or control in A549/DDP or A549 cell lines. (**b**) Flow cytometric analysis of cell cycle in A549/DDP and A549 were determined after transfected miR-181b mimics, miR-controls, miR-181b inhibitors or negative controls combined with DDP. (**c**) Flow cytometric analysis of apoptosis in A549/DDP and A549 were determined after transfected miR-181b mimics, miR controls, miR-181b inhibitors or negative controls combined with DDP. *P < 0.05; **P < 0.01; ***P < 0.001.

Additionally, The Supplementary Information published with this Article contains an error in Figure S1C, where the images representing the “anti-NC” group and the “Untreated” group are the same, and the image representing the “anti-181b” is incorrect.

The correct Supplementary Information is provided below.

## Supplementary Information

Supplementary Information.

