# Supplementary material for: MiR-181b regulates cisplatin chemosensitivity and metastasis by targeting TGFβR1/Smad signaling pathway in NSCLC
Source: Sci Rep. 2015 Dec 1;5:17618. doi: 10.1038/srep17618 (PMC4664936; doi:10.1038/srep17618)
Supplement: Supplementary Information [file srep17618-s1.doc]

**MiR-181b regulates cisplatin chemosensitivity**

**and metastasis by targeting TGFβR1/Smad signaling pathway in NSCLC**

Xiaoyuan Wang^1*^, Xuesong Chen^1^, Qingwei Meng^1^, Hu Jing^1^, Hailing Lu^1^, Yanmei Yang^2^, Li Cai^1§^, Yanbin Zhao^1§^

**Method**

**Patients and tissue samples**

Each patient had signed informed consent for medical record review and tissue sample donation. This study was approved by the institutional review board at Harbin Medical University and conducted according to all current ethical guidelines. Fresh tumor tissues from 38 patients with NSCLC were obtained at the Department of Thoracic Surgery of the Affiliated Tumor Hospital of Harbin Medical University between Oct 2008 and August 2009. The patients were follow-up until Dec, 2013. Histopathologic subtypes were determined according to the WHO classification. The International Staging System was used for pathological staging. Tumor tissue collected at the time of curative surgical resection was stored in RPMI-1640 for primary culture of NSCLC cells and subsequent cell proliferation assay within 30 minutes. Long-term storage of the tissues was at -80°C. At the time of surgery and tissue collection, none of the patients had received neoadjuvant chemotherapy or radiation therapy.

**TUNEL assay**

TUNEL assay was performed in sections using an In Site Cell Death Detection Kit (Roche Diagnostics GmbH, Mannheim, Germany) principally according to the manufacturer's instructions. The sections of lung tissues were incubated with the TUNEL reaction mixture for 1 hr at 37°C in a dark, humidified chamber. Labeled DNA was visualized with an anti-fluorescein antibody conjugated with peroxidase (POD) using 3,3′-diaminobenzidine (DAB) as the chromogen. Sections were then washed, counterstained with hematoxylin. For negative control, TdT was omitted from the reaction mixture. 5 high-power fields (×400) were randomly selected, and the number of apoptotic cells was counted for each field. Apoptosis index (AI) = number of positive cells/number of total cells. According to the manufacturer's instructions, the staining results are interpreted by pathologists.

**Supplementary legends**

**Fig. S 1 MiR-181b inhibits cell proliferation in NSCLC cells.**

a qPCR assay indicated that expression level of miR-181b in H1650 and transfected cells.

b CCK analysis of cell proliferation after transfected with miR-181b inhibitors, or control in H1650 cell lines.

c Colony-forming efficiency was detected in H1650 cell lines transfected with miR-181b inhibitors or control. **P*<0.05; ***P*<0.01.

**Fig. S 2 MiR-181b enhances chemosensitivity of NSCLC cells to DDP.**

a CCK analysis of IC50 values of DDP after transfected with miR-181b inhibitors or control in H1650 cell lines. **P*<0.05; ***P*<0.01; ****P*< 0.001.

**Fig. S 3 MiR-181b attenuates scratch, migration and invasion of lung cancer cells.**

a Wound-healing assay in H1650 cell lines were determined after transduction with the miR-controls or miR-181b inhibitors or negative controls.

b Transwell migration and invasion assays for H1650 cell lines were determined after transduction with the miR-controls, or miR-181b inhibitors or negative controls. **P*<0.05; ***P*<0.01; ****P*< 0.001.

**Fig. S 4 TGFβR1 is a direct target of miR-181b.**

a qRT-PCR and western blot detection of TGFβR1 mRNA and protein expression in H1650 cells transfected with anti-miR-181b. U6 or GAPDH was used as an internal control. **P*<0.05; ***P*<0.01; ****P*< 0.001.


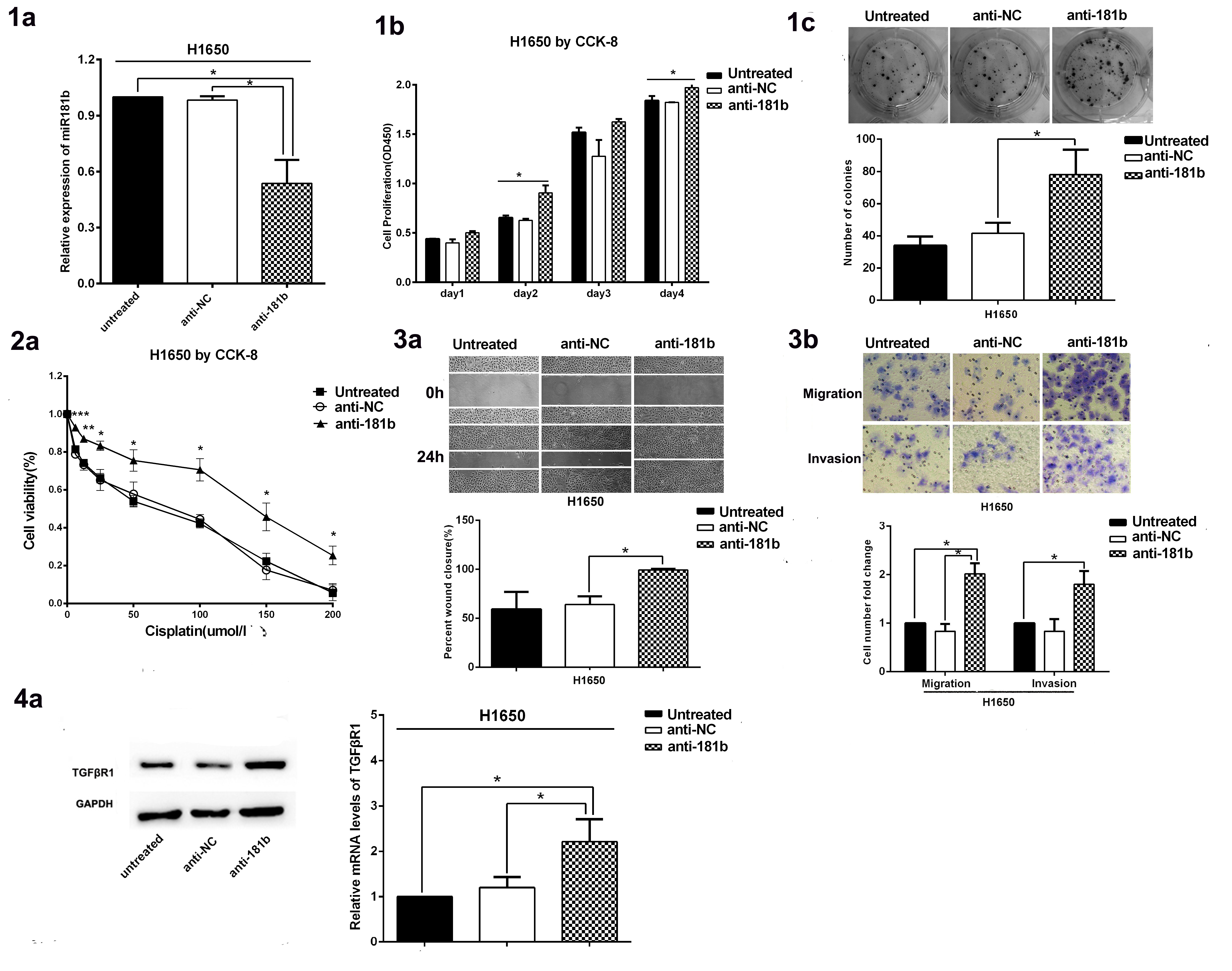


Supplementary table 1. Association between miR181b expressions and clinicopathologic factors in patients with NSCLC

| Variable | No. of Patients | miR181b expression | | *P* (χ^2^) | TGFβR1 Expression | | | | Positive  Rate(%) | *P* (χ^2^) |
| --- | --- | --- | --- | --- | --- | --- | --- | --- | --- | --- |
|  |  | High(n) | low(n) |  | (-) | (+) | (++) | (+++) |  |  |
| All cases | 38 | 21 | 17 |  | 17 | 6 | 8 | 7 |  |  |
| Age (y) |  |  |  | 0.461 |  |  |  |  |  | 0.488 |
| <60 | 26 | 15 | 11 |  | 11 | 3 | 7 | 5 | 57.69 |  |
| ≥60 | 12 | 6 | 6 |  | 6 | 3 | 1 | 2 | 50.00 |  |
| Gender |  |  |  | 0.210 |  |  |  |  |  | 0.705 |
| male | 23 | 11 | 12 |  | 12 | 3 | 4 | 4 | 47.83 |  |
| female | 15 | 10 | 5 |  | 5 | 3 | 4 | 3 | 66.67 |  |
| Differentiation |  |  |  | 0.135 |  |  |  |  |  | 0.125 |
| Well | 14 | 8 | 6 |  | 6 | 1 | 3 | 4 | 57.14 |  |
| moderate | 7 | 6 | 1 |  | 1 | 1 | 2 | 3 | 85.71 |  |
| poor | 17 | 7 | 10 |  | 10 | 4 | 3 | 0 | 41.18 |  |
| Tumor size (cm) |  |  |  | **0.015** |  |  |  |  |  | 0.244 |
| ≤3 | 12 | 9 | 3 |  | 4 | 3 | 3 | 2 | 66.67 |  |
| 3~5 | 21 | 13 | 8 |  | 8 | 3 | 5 | 5 | 61.90 |  |
| >5 | 5 | 0 | 5 |  | 5 | 0 | 0 | 0 | 0.00 |  |
| pT status |  |  |  | **0.013** |  |  |  |  |  | 0.729 |
| pT1 | 11 | 10 | 1 |  | 3 | 3 | 3 | 2 | 72.73 |  |
| pT2 | 26 | 11 | 15 |  | 13 | 3 | 5 | 5 | 50.00 |  |
| pT3 | 1 | 0 | 1 |  | 1 | 0 | 0 | 0 | 0.00 |  |
| pN stage |  |  |  | 0.508 |  |  |  |  |  | **0.011** |
| pN0 | 27 | 16 | 11 |  | 15 | 6 | 4 | 2 | 44.44 |  |
| pN1 | 3 | 2 | 1 |  | 1 | 0 | 0 | 2 | 66.67 |  |
| pN2 | 8 | 3 | 5 |  | 1 | 0 | 4 | 3 | 87.50 |  |
| pTNM stage |  |  |  | **0.034** |  |  |  |  |  | 0.071 |
| I | 23 | 16 | 7 |  | 11 | 5 | 4 | 3 | 52.17 |  |
| II | 7 | 1 | 6 |  | 5 | 1 | 0 | 1 | 28.57 |  |
| III | 8 | 4 | 4 |  | 1 | 0 | 4 | 3 | 50.00 |  |
| Histological classification |  |  |  | 0.460 |  |  |  |  |  | 0.170 |
| Squamous cell carcinoma | 10 | 7 | 3 |  | 3 | 2 | 1 | 4 | 70.00 |  |
| Adenocarcinoma | 28 | 14 | 14 |  | 14 | 4 | 7 | 3 | 50.00 |  |
| DDP sensitivity |  |  |  | **0.046** |  |  |  |  |  | **0.011** |
| Sensitive | 20 | 8 | 12 |  | 12 | 5 | 2 | 1 | 40.00 |  |
| Resistant | 18 | 13 | 5 |  | 5 | 1 | 6 | 6 | 72.22 |  |

Supplementary table 2. Prognostic factors for NSCLC patients

| Variables | HR | Univariate  95% CI | *P* | HR | Multivariate  95% CI | *P* |
| --- | --- | --- | --- | --- | --- | --- |
| OS |  |  |  |  |  |  |
| Age (year) |  |  |  |  |  |  |
| <60 vs. ≥60 | 0.914 | 0.242-3.446 | 0.894 |  |  |  |
| Gender |  |  |  |  |  |  |
| Male vs. female | 0.129 | 0.016-1.010 | 0.051 |  |  |  |
| Differentiation |  |  |  |  |  |  |
| Well vs moderate/poor | 0.719 | 0.334-1.548 | 0.399 |  |  |  |
| Tumor size (cm) |  |  |  |  |  |  |
| ≤5 vs >5 | 2.601 | 0.687-9.852 | 0.159 |  |  |  |
| pT status |  |  |  |  |  |  |
| pT2-4 vs. pT1 | 4.320 | 0.552-33.803 | 0.163 |  |  |  |
| pN stage |  |  |  |  |  |  |
| pN1-2 vs. pN0 | 3.580 | 1.089-11.771 | **0.036** | 1.809 | 0.347-9.429 | 0.482 |
| pTNM stage |  |  |  |  |  |  |
| II-III vs. I | 5.067 | 1.342-19.140 | **0.017** | 3.194 | 0.403-25.306 | 0.271 |
| Histological classification |  |  |  |  |  |  |
| Adenocarcinoma/ vs. Squamous cell cancer | 2.034 | 0.432-9.572 | 0.369 |  |  |  |
| miR181b expression |  |  |  |  |  |  |
| low vs. high | 0.157 | 0.034-0.732 | **0.018** | 0.022 | 0.001-0.328 | **0.006** |
| TGFβR1 expression |  |  |  |  |  |  |
| TGFβR1 (+) vs. TGFβR1 (-) | 0.251 | 0.066-0.951 | **0.042** | 11.334 | 0.869-147.754 | 0.064 |

Supplementary table 3. Correlation of TGFβR1 and prognosis, metastasis in lung cancer in the Oncomine online Database (www.oncomine.org)

|  | Fold change | P value | Database (case number) |
| --- | --- | --- | --- |
| Dead at 1 year vs alive at 1 year in lung adenocarcinoma | 1.971 | 1.72E-4 | Hou Lung Statistics(8) |
| Dead at 3 year vs alive at 3 year in lung adenocarcinoma | 1.704 | 2.36E-4 | Hou Lung Statistics(9) |
| Dead at 5 year vs alive at 5 year in lung adenocarcinoma | 1.594 | 0.001 | Hou Lung Statistics(44) |
| Recurrence at 1 year vs no recurrence at 1 year in lung adenocarcinoma | 1.814 | 0.001 | Okayama Lung Statistics(284) |
| Dead at 5 year vs alive at 5 year in lung adenocarcinoma | 2.009 | 1.91E-4 | Okayama Lung Statistics(993) |
| Dead at 3 year vs alive at 3 year in lung adenocarcinoma | 1.735 | 0.006 | Okayama Lung Statistics(1421) |
| Recurrence at 5 year vs no recurrence at 5 year in lung adenocarcinoma | 1.812 | 8.52E-5 | Okayama Lung Statistics(1541) |
| Metastasis vs primary site | 2.053 | 8.41E-6 | Bhattacharjee Lung Statistics(168) |
| Metastasis vs primary site in lung cancer | 1.503 | 0.046 | Garber Lung Statistics (758) |
